# Pleural clinic: where thoracic ultrasound meets respiratory medicine

**DOI:** 10.3389/fmed.2023.1289221

**Published:** 2023-10-11

**Authors:** Mariaenrica Tinè, Matteo Daverio, Umberto Semenzato, Elisabetta Cocconcelli, Nicol Bernardinello, Marco Damin, Marina Saetta, Paolo Spagnolo, Elisabetta Balestro

**Affiliations:** Department of Cardiac, Thoracic, Vascular Sciences and Public Health, University of Padova, Padova, Italy

**Keywords:** pleura, thoracic ultrasound, pleural effusion, unmet clinical need, medical thoracoscopy

## Abstract

Thoracic ultrasound (TUS) has become an essential procedure in respiratory medicine. Due to its intrinsic safety and versatility, it has been applied in patients affected by several respiratory diseases both in intensive care and outpatient settings. TUS can complement and often exceed stethoscope and radiological findings, especially in managing pleural diseases. We hereby aimed to describe the establishment, development, and optimization in a large, tertiary care hospital of a pleural clinic, which is dedicated to the evaluation and monitoring of patients with pleural diseases, including, among others, pleural effusion and/or thickening, pneumothorax and subpleural consolidation. The clinic was initially meant to follow outpatients undergoing medical thoracoscopy. In this scenario, TUS allowed rapid and regular assessment of these patients, promptly diagnosing recurrence of pleural effusion and other complications that could be appropriately managed. Over time, our clinic has rapidly expanded its initial indications thus becoming the place to handle more complex respiratory patients in collaboration with, among others, thoracic surgeons and oncologists. In this article, we critically describe the strengths and pitfalls of our “pleural clinic” and propose an organizational model that results from a synergy between respiratory physicians and other professionals. This model can inspire other healthcare professionals to develop a similar organization based on their local setting.

## Introduction

Pleural diseases constitute a highly prevalent yet often under-recognized cluster of conditions, encompassing pleural effusion, thickening, and abnormalities. By its double layer of mesothelial cells, the pleura actively produces substances that maintain the delicate balance between fluid production and reabsorption in the pleural space. In the United States alone, approximately 1.5 million cases of pleural effusion are diagnosed annually. Pleural effusion is a secondary phenomenon in various thoracic and extra-thoracic disorders, including heart and kidney failure, pancreatitis, pneumonia and lung cancer (1). Additionally, pleural effusion and thickening can frequently result from conditions directly affecting the pleura, such as pleural infections, asbestos plaques and mesothelioma.

Furthermore, several relatively uncommon conditions, including connective tissue diseases and auto-inflammatory disorders can incite pleural reactions (2). Therefore, effectively dealing with and managing pleural diseases necessitates a combination of experience, skills, training, and access to appropriate diagnostic tools to discern the underlying cause of pleural reaction. Traditional imaging techniques serve as sensitive and specific means for determining the presence, extent, and potential etiology of pleural diseases. For instance, chest X-ray can detect fluid collections ranging between 250 and 600 mL (3). Such a wide range is due to the relative inability of standard X-ray to detect pleural fluid if obtained in the supine position, when the liquid is evenly distributed, or only in one projection if the fluid volume is small. On the other extreme, chest CT can detect a minimal amount of pleural fluid and irregularities and, when completed with contrast medium – both iodine and 18-FDG – it facilitates the differentiation between vascularized plaques and benign thickening (4). Following conventional radiologic techniques, thoracic ultrasound (TUS) has revolutionized the management of pleuro-pulmonary diseases. TUS can be easily performed bedside and does not entail exposure to ionizing radiation, rendering it a rapid and safe tool for evaluating several respiratory conditions (5). When conducted by experienced physicians, TUS surpasses the performance of chest X-ray in diagnosing pulmonary edema and pneumothorax and reaches a sensitivity level of 90–97% and a 94–99% specificity in diagnosing pneumonia (6, 7). It is precious in detecting pleural effusion, as it can identify even small fluid volumes (5–20 mL) (8) and characterize loculations of complex fluid collections.

Consequently, TUS guides thoracentesis, chest tube placement, and thoracoscopy (9). Using tissue movement and deformation analysis, TUS can also predict entrapped lung in malignant pleural effusion, aiding in the decision-making process between attempting pleurodesis or opting for an indwelling pleural catheter (10). Indeed, TUS has been widely applied in different settings from critical care units for acute onset conditions to outpatient clinics for disease screening and follow-up (11, 12).

TUS’s feasibility, safety and reproducibility have inspired the creation of the so-called “pleural clinic” or “pleural unit.” Typically located within tertiary care hospitals, these units serve as unique centers for patients with various pleural disorders, including recurrent pleural effusion and nonspecific pleuritis, referred from peripheral hospitals. According to Hooper et al., the ideal pleural clinic would be led by an expert “pleurologist,” defined as a “*pulmonologist with extended experience and training in the management of pleural disease*” (13) with regular access to pleural procedures facilities, pathology and radiology service, and to thoracic surgeons’ consultation. Upon admission to such a pleural service – whether termed a unit or clinic (14–16) – patients undergo a comprehensive clinical evaluation and, through a judicious combination of imaging technique, TUS, and pleural procedure, they receive an accurate diagnosis and tailored management. Experience and training in TUS were been considered “*useful additional skills*” since 2012 when Sura et al. reported that combining a pleurologist, TUS expertise, and a dedicated procedure room significantly improves pleural disease management (17). As described, the pleural service shortened patient waiting lists, and length of stay, avoided inappropriate emergency room admissions and reduced radiologic procedures, leading to significant financial savings (17). To further optimize pleural disease management, the pleural team might include a dedicated nurse who provides essential support to the chest physician and respiratory residents assist in data collection and gain access to TUS training (18). Implementing such coordinated service might be challenging, especially in peripheral hospitals. In light of the heterogeneous demand and possibility, Evison and colleagues stratified the minimum requirement of a pleural service into 3 categories: the first one can perform basic pleural diagnostic and is directly connected to category 2, a service led by an expert pleural operator who led a pleural clinic with dedicated nursing support but does not reach the levels of category 3 service where advanced diagnostics and therapeutics (anesthetic thoracoscopy and indwelling pleural catheter insertion) are available and complex cases are referred to (19).

Potential obstacles to setting up a pleural service include (1) a lack of networking among professionals, who might be hesitant to refer patients elsewhere or may be unaware of the service’s existence, (2) insufficient resources to establish a dedicated operating room with essential equipment or to recruit the appropriate staff, and (3) prioritization of urgent/different clinical services (13).

These challenges, among others, can considerably limit the efficiency and effectiveness of a pleural service. In this article, we provide a detailed account of the evolution of a pleural service at our hospital, elucidating its growing significance, the strategies we employed to surmount obstacles, and the ongoing limitations we contend with.

## The context

Italy’s National Health Service is a public system established in 1978 that guarantees residents free or low-cost healthcare. Each regional government is responsible for resource management, with relative heterogeneity in treatment standards across the country. The Azienda Ospedale-Università di Padova (AOUP), a University Hospital in the Veneto region, is a referral center for hospitals in northern Italy, offering tertiary care in a “hub and spoke” model. An experienced thoracic surgery and pathology unit cooperates with oncologists from the Istituto Oncologico Veneto in diagnosing lung cancer. In this context, the pulmonary medicine unit plays a pivotal role in expediting the diagnosis of thoracic cancers. Respiratory patients receive evaluations in the outpatient clinic and, when pleural disorders are suspected, are referred to the pleural service, closely linked to the endoscopy unit where pleural procedures are performed.

## The pleural service birth and development: a bildungsroman

The respiratory endoscopy unit at AOUP has long played a crucial role in diagnosing and managing airway and pleural diseases. In the context of pleural effusions, it conducts approximately 100 medical thoracoscopies annually. Medical thoracoscopy, or pleuroscopy, is a minimally invasive procedure that, under light sedation, allows the evaluation of the pleural space, effusion drain, sampling of parietal pleura, and poudrage talc pleurodesis, with a diagnostic yield of 90–100% (20). In cases where the effusion is neoplastic, patients are appropriately referred to either the Oncology service or palliative care. Benign effusions are managed according to their etiology: tubercular effusion is treated with the infectious disease specialist, whereas abscess and empyema are treated with systemic antibiotics and, when necessary, local fibrinolytic. Nevertheless, in a significant percentage of patients – ranging from 6 to 31% (21) – biopsies reveal non-specific pleurisy, a “non-diagnosis” that raises more questions than answers. Studies have demonstrated that non-specific pleurisy has the potential to become malignancy in 1 over 6 cases within 21 months (21) and often leads to recurrent pleural effusion, sometimes even contralateral, underscoring the imperative for rigorous follow-up of these patients. To address this subset of patients and capitalize on TUS’s increasing availability, we opted to establish a dedicated outpatient clinic. Key components of our “pleural service” included the presence of pulmonologists with expertise in pleural disease management who alternated between the endoscopy unit and the outpatient clinic, medical trainees proficient in TUS who collected data, and the availability of an ultrasound machine. Non-specific pleuritis patients underwent a personalized follow-up program tailored to baseline radiologic findings, individual risk factors and clinical conditions. This program included at least one TUS examination every 3 to 6 months to detect pleural effusion recurrence promptly and provide aspiration if needed. Subsequently, the clinic’s focus expanded to encompass the follow-up of patients who had undergone pleural procedures, enabling the prompt assessment and management of complications and addressing pleurodesis failure.

**Figure 1:**
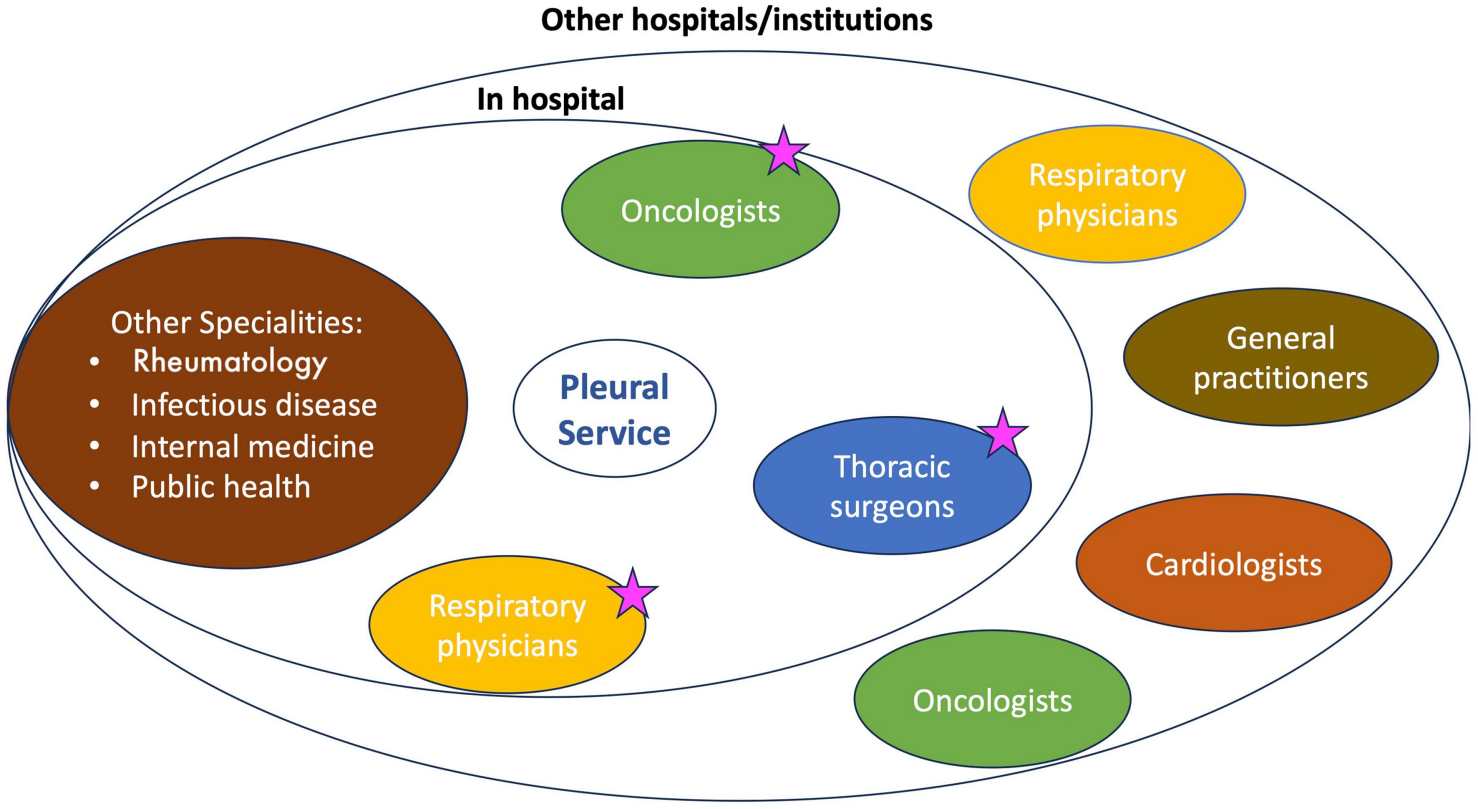
The pleural service stakeholders. The ambulatory pleural service provides examinations and basic procedures for patients referred from both in-hospital specialists and other hospitals/institutions. The interaction between in-hospital specialists is key to patient management. Starred professionals are part of the oncologic multidisciplinary team, along with radiologists and pathologists.

The pleural service was rapidly acknowledged within the hospital and throughout the Veneto region, resulting in a significant upsurge in referred cases (Figure 1).

Since 2018, the number of referrals has steadily risen from 2 to 3 per week to over 10, totaling 1,020 outpatient evaluations until March 2023 (Supplementary Figure S1).

Over time, the range of procedures and indications expanded, and the pleural service evolved to meet the needs. Initially, referrals primarily came from respiratory physicians within our hospital, who, upon identifying patients with potential pleural disorders, directed them to us for the most suitable diagnostic and therapeutic interventions. Subsequently, oncologists emerged as our primary source of patients. In cases of unilateral effusion, we initiate the evaluation at the ambulatory pleural service and review the patient’s medical history. When feasible, we recommend medical thoracoscopy, a procedure conducted in the endoscopy room under light sedation. This approach ensures ample tissue for pathology characterization, may be therapeutic and allows performing pleurodesis, thus reducing the risk of pleural effusion recurrence. Notably, TUS can effectively guide medical thoracoscopy (Supplementary Figure S2) even without pleural fluid, ensuring precise identification of thoracic lesions (22). If technically unfeasible, we seek thoracic surgeons’ consultation but, due to a considerably long waiting time, we might perform a parallel procedure to get a diagnosis (i.e., endoscopy, transthoracic biopsy) and alleviate pleural effusion-related symptoms through thoracentesis, usually in a day hospital setting. In contrast, when the pleural effusion etiology is clear (e.g., mesothelioma), acute symptoms can be effectively alleviated through fluid aspiration, a procedure that any physician can perform. However, in an emergency setting, the analyses on pleural fluid may be overlooked, and complementary therapeutic measures, including pleurodesis, might be missed. In response to this concern, oncologists have taken proactive measures to prevent excessive pleural fluid accumulation in their patients. They regularly refer patients with small effusions to our combined follow-up program: TUS examinations, performed every 3 months, are alternated with CT scans, also generally performed every 3 months, resulting in a 45 day-time frame in which fluid can accumulate. When the effusion causes symptoms and imaging confirms its increase, a chest tube insertion or a thoracentesis, as appropriate, is scheduled at our unit.

This “alternate strategy” proves effective when a strong collaboration between pulmonologists and oncologists is established.

**Figure 2:**
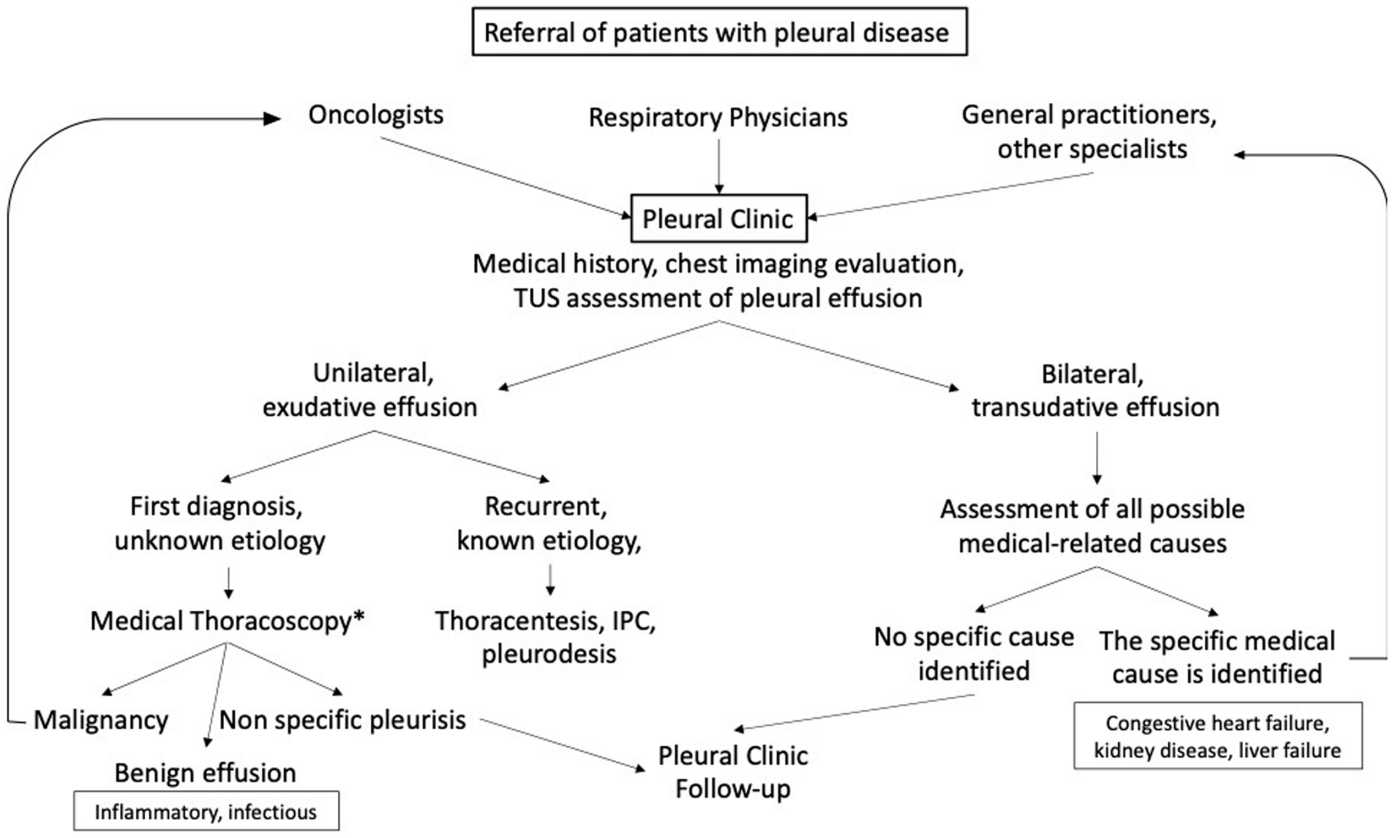
The pleural clinic workflow. * when medical thoracoscopy is not feasible, we seek thoracic surgeons’ collaboration. IPC, indwelled pleural catheter.

In contrast, referrals from other specialists are typically unidirectional, and once a diagnosis is confirmed, the patient is redirected to the proper specialist. When a transudate is detected, potential medical causes are investigated, and the patient is referred to the cardiologist, nephrologist or gastroenterologist, as appropriate (Figure 2).

We encountered a few cases of transudative effusions recurring despite appropriate management. Moreover, we observed a few patients with significant cardiac comorbidities whose unilateral transudative effusion recurred evolving into an exudative effusion over time. Unfortunately, those patients are frequently ineligible for medical thoracoscopy thus they are evaluated by imaging, and provided medical supportive care, including diuretics, steroids and long-term oxygen therapy. Supplementary Table S1 provides an overview of the most frequent pleural conditions with their corresponding management.

### TUS approach

By conducting rapid examination of target areas and comparing the affected side with the healthy side, TUS enables the detection and monitoring of even minute fluid accumulation and various other pleuro-parenchymal abnormalities. We routinely store data on an external device for easy reference and generate printed copies of the most significant ultrasound images for sharing with both patient and referring doctor. With advancements in new ultrasound machines, storing higher-quality outputs facilitates swift comparisons with previous images. Our scanning protocol typically includes the use of both linear and convex probes for assessing the pleural surface and evaluate the presence of fluid, respectively. Doppler ultrasound is frequently employed to assess vascularization in subcutaneous nodules or blood collection that may develop after pleural procedures. In addition, M-mode is often necessary to evaluate pleural sliding. Another pivotal component of an effective pleural service is maintaining a short waiting list to ensure prompt management of pleural diseases, which, if left untreated, can progress rapidly.

## Pleural service: further indications of a TUS-based follow up

In addition to its crucial role in the emergency room assessment of patients with respiratory symptoms/signs (23), TUS examination can be helpful, albeit not exhaustive, for detection and follow-up of a variety of lung abnormalities (24). Although the study of interstitial lung diseases (ILDs) requires high-resolution CT scan, the complementary assessment using lung US can provide safe and reproducible data during follow-up. Fibrotic changes cause the so called “B-lines,” vertical artifacts that correlate with lung fluid, presence and extent of ILDs, lung contusions and COVID-19 (25). Ongoing studies are evaluating protocols and new US applications for distinguishing fibrotic B-lines (26). In patients with rheumatoid arthritis, US can accurately detect ILDs, and the number of B-lines has been associated with the degree of functional impairment and the levels of circulating inflammatory markers (27). The sensitivity and specificity of TUS in comparison to HRCT are currently under investigation (28). Similarly, US assessment has been effectively employed as a diagnostic tool for COVID-19 pneumonia, given the predominant involvement of the lung periphery (29). In the late phase of infection, lung US features correlate with oxygen requirements and chest X-ray consolidation (30) suggesting that lung US can serve as a substitute for standard imaging in the follow-up of a number of conditions as well as in intensive care setting (31). Of particular interest is the development of point-of-care ultrasound (POCUS) protocols for the global assessment of COVID-19 patients, especially in critical conditions. In such disease, TUS are effectively integrated to evaluate all the possible consequences of SARS-CoV-2 infection, including cardiac dysfunction, parenchymal involvement and thrombotic complications (32). Moreover, lung US can assist in diagnosing supleural pneumonia and its complications and can be employed for the follow-up of patients with pulmonary infarction. In this latter case, the US may reveal ongoing resolution even before contrast enhanced chest CT (25).

Another remarkable application of US is in the assessment and follow-up of diaphragmatic dysfunction in chronic obstructive pulmonary disease (COPD) patients. Building upon data from mechanical ventilation weaning (33), US can be used to assess diaphragm mobility and thickness (34), thereby offering valuable guidance for targeted rehabilitation programs (35).

## Is the pleural service worth the effort?

The necessity for a specialized setting to assess pleural disorders and conduct pleural procedures has long been advocated. Available literature provides indications and suggestions that are mainly shaped on the UK health system needs and resources (13, 16, 18, 36) since British respiratory physicians are among the leaders in pleural disorders and their guidelines are applied worldwide [see (1, 37, 38)]. In Italy, we faced the growing need for a pleural team, with a dedicated outpatient clinic and a group of physicians who could manage this subset of thoracic disorders on a multidisciplinary level. As suggested by Evison et al., the basic requirements of a pleural service are an expert in TUS and pleural disease and a “good enough” US machine (19). The use of US has revolutionized the medical approach to a range of thoracic diseases, providing a safe, repeatable, and rapid tool to assess the chest and exclude/confirm the presence of peripheral pulmonary lesions, pleural effusion and other abnormalities. Its application was even widened during the COVID-19 pandemic, with respiratory physicians and non-specialists using US to quickly assess patients suspected of COVID-19 pneumonia (30). For instance, US can detect B line pattern, pneumonia, and deep venous thrombosis, all frequent complications of the SARS-CoV2 infection.

Nevertheless, managing pleural diseases remains a complex issue requiring specialized care. Moreover, the pandemic posed a significant threat to patients with pleural diseases, primarily due to the limited access to hospitals and the reduced number of clinicians dedicated to the care of pleural disease. Nonetheless, we reinforced protective measures and utilized facilities from oncology and other specialist services that were less involved in COVID-19 management. With these precautions, following an initial decline in the number of procedures performed, we could maintain a standard level of service.

Among other common hurdles described in the literature and summarized in Supplementary Table S2, we encountered additional challenges, including (1) the requirement for a larger, well-equipped ambulatory room to conduct minor procedures like thoracentesis, medications and slurry pleurodesis and (2) the necessity for a dedicated nurse or team of nurses who can assist with venous access and other basic procedures.

Meeting these requirements would enable the concurrent performance of simple procedures alongside the endoscopy room, which could be reserved for medical thoracoscopy, indwelling pleural catheter and large-bore chest tube insertion. This approach would help reduce waiting lists and optimize the allocation of staff and resources. The shortage of available beds is another common issue. To address this, we optimized hospital stays by ensuring a swift turnover by prescribing pre-admission examinations including blood tests and ECG, and planning post-discharge follow-up visits. Moreover, by fostering close collaboration with other hospitals and wards, we aim to minimize the length of patient stays until chest tube removal. Whenever possible, we perform procedures such as thoracentesis and IPC in a day hospital setting, ensuring appropriate post-procedural monitoring (Supplementary Figure S3).

A frequently described problem, which we have yet to encounter, is the hesitation of our colleagues in referring patients to our service (13). Frequently, we examine patients with significant hemithorax opacities that hide atelectasis or hemidiaphragm elevation misclassified as pleural effusions. Not uncommonly, we also assess patients with known liver/kidney or heart failure and bilateral effusion who only require optimization of their medical therapy. Thoracic surgeons may also refer to our service patients with pleural disease, particularly when a follow-up is needed, thereby recognizing our expertise and proficiency in TUS. In addition, we work in a setting comprising expert pathologists, oncologists, and thoracic surgeons who meet weekly to discuss the appropriate management of challenging patients.

Despite these potential limitations, setting up a dedicated pleura service has the potential to reduce charges for pleural disease care, shorten hospital stay, minimize diagnostic delay, and secure early recognition of post procedural complications or disease recurrence (36, 39). In our experience, the pleural service team works synergically with the pulmonary interventional team to define priorities and the proper setting of any planned procedures. Moreover, by documenting TUS findings during each visit, respiratory physicians can discuss challenging cases, offering valuable educational opportunities for resident doctors. All data are securely stored and could serve as the foundation for clinical studies to elucidate disease pathogenesis and optimize treatments.

## Conclusion

In conclusion, the availability of human and technical resources is crucial for establishing a service entirely committed to the timely diagnosis and management of pleural disease. In expert hands, thoracic ultrasound can safely and effectively identify the problem, suggest the best setting to manage it, and provide guidance for diagnostic/therapeutic procedures. Access to the oncologic multidisciplinary team, as well as networking with clinicians from other specialties, including rheumatologists and infectious disease specialists, are vital aspects to consider for the successful setting up of a pleural service.

## Data availability statement

The original contributions presented in the study are included in the article/Supplementary material, further inquiries can be directed to the corresponding author.

## Author contributions

MT: Conceptualization, Writing – original draft. MatD: Data curation, Writing – original draft. US: Conceptualization, Writing – original draft. EC: Writing – review & editing. NB: Writing – review & editing. MarD: Investigation, Supervision, Writing – review & editing. MS: Supervision, Writing – review & editing. PS: Supervision, Writing – review & editing. EB: Conceptualization, Supervision, Writing – review & editing.
