# Supplementary figures and images for: Pleural clinic: where thoracic ultrasound meets respiratory medicine
Source: Front Med (Lausanne). 2023 Oct 11;10:1289221. doi: 10.3389/fmed.2023.1289221 (PMC10598727; doi:10.3389/fmed.2023.1289221)

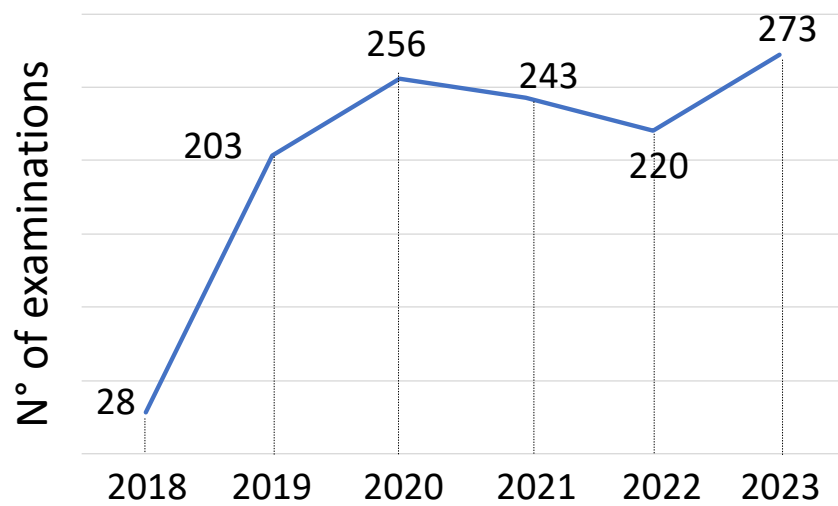

Supplement: Supplementary file 1 [file Image_1.pdf]

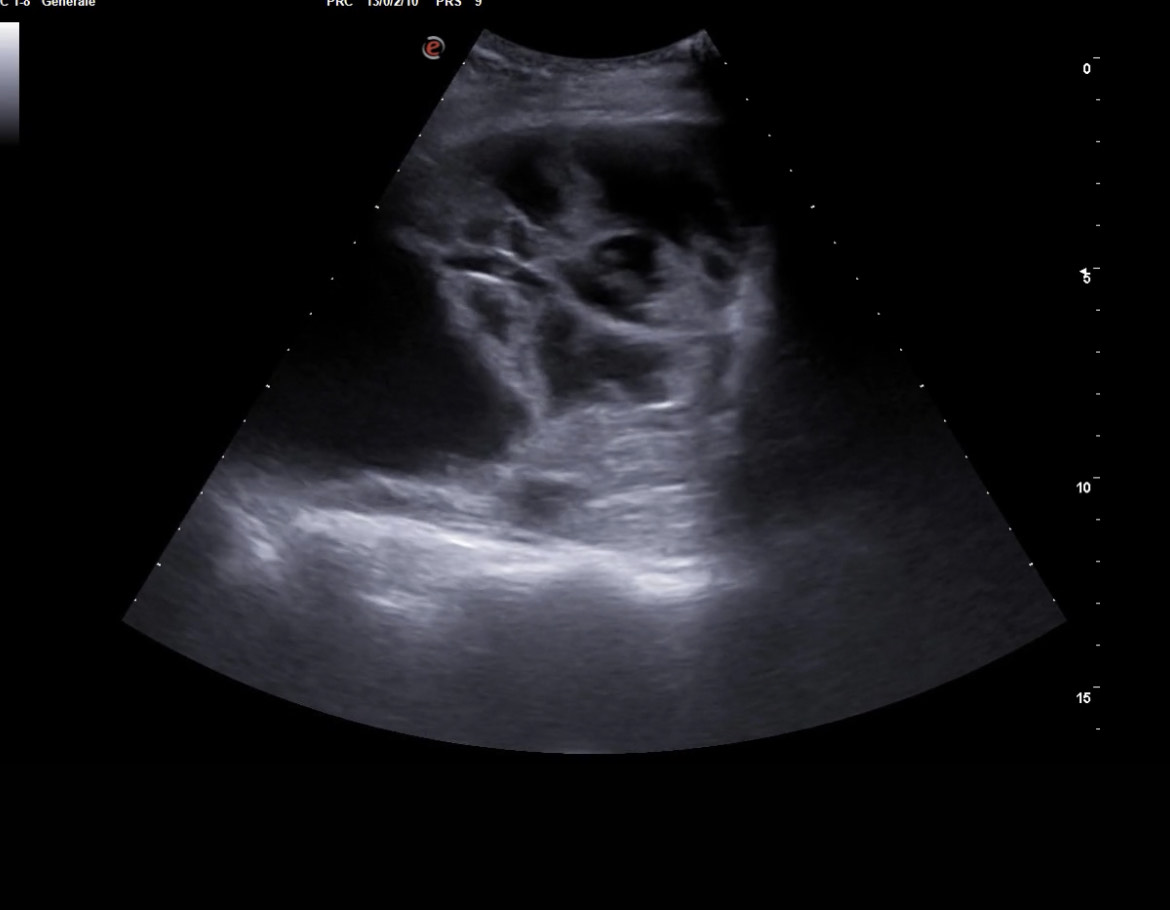

Supplement: Supplementary file 2 [file Image_2.jpeg]

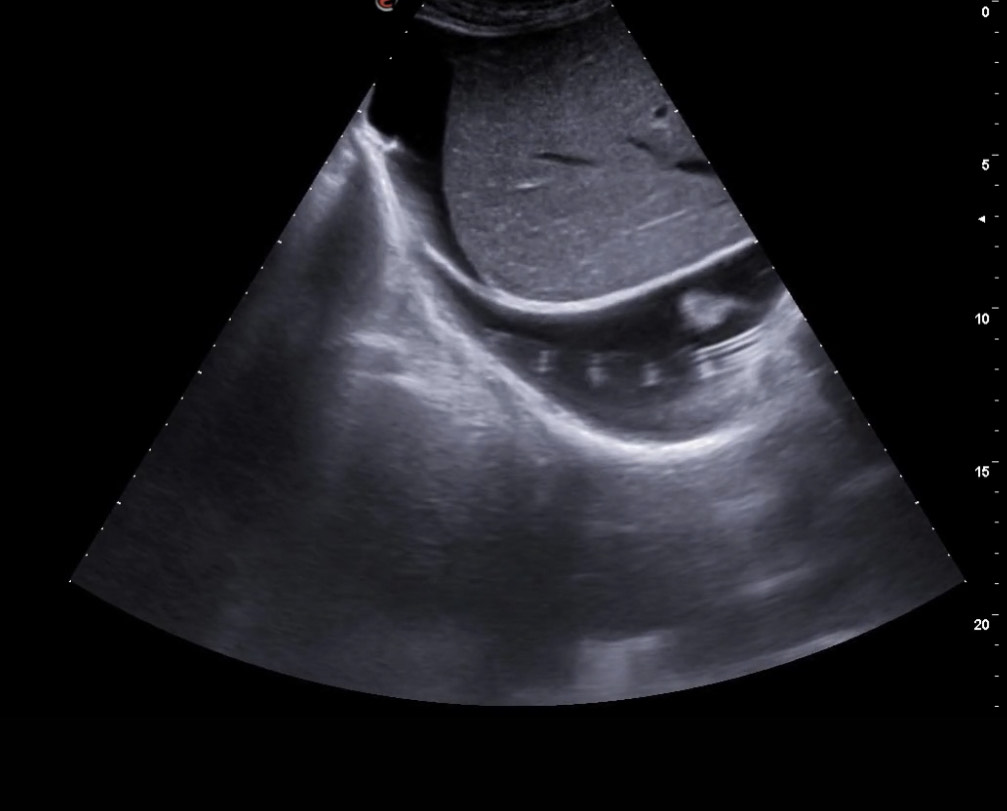

Supplement: Supplementary file 3 [file Image_3.jpeg]
