# Supplementary material for: Pleural clinic: where thoracic ultrasound meets respiratory medicine
Source: Front Med (Lausanne). 2023 Oct 11;10:1289221. doi: 10.3389/fmed.2023.1289221 (PMC10598727; doi:10.3389/fmed.2023.1289221)
Supplement: Supplementary file 4 [file Data_Sheet_1.docx]

**Table S1. Possible management strategies for pleural diseases**

| **Which problem?** | **How we manage it?** | **Follow-up or discharge?** |
| --- | --- | --- |
| Bilateral effusion | Consider all medical-related etiology | Referral to cardiology, nephrology or liver disease service |
| Undiagnosed unilateral pleural effusion | Diagnostic work-up according to risk-benefit ratio and patients’ wishes | Malignant effusion 🡪 oncology referral or MDD  Benign effusion 🡪 referral to infectious disease or rheumatology service  Nonspecific pleuritis 🡪 follow-up |
| Recurrent pleural effusion | Repeated thoracentesis  Chest tube and pleurodesis  Indwelled pleural catheter | Follow-up at PS  Home health care |
| Pleural thickening and plaques | Contrast medium CT and PET-CT scan  Transthoracic biopsy if suspicious | Follow-up at PS  Malignancies 🡪 oncology referral |
| Empyema | Large-bore chest tube placement, systemic and local antibiotics, thoracic surgeon cooperation when needed | Follow up at PS to assess resolution and manage risk factors for recurrency |
| Peripheral abscess | Referral to interventional radiologist | Follow up at PS to assess resolution and manage risk factors for recurrency |

MDD: multidisciplinary discussion. PS: pleural service. CT: computed tomography. PET: positron emission tomography.

**Table 2. Pro and cons of developing a pleural service**.

| **PRO** | | **CONS** | |
| --- | --- | --- | --- |
| **Advantages** | **Potential benefits** | **Disadvantages** | **Potential solutions** |
| Reduced ER admissions | Reduced health costs | Requires experienced staff | Discuss the advantages, promote resources allocation |
| Shortened time to diagnosis |  | Requires proper space and setting | Make alliances with oncologists or other PS stakeholders |
| Improved pleural disease management |  | Procedural complications require direct access to respiratory ward | Dedicated bed(s) for interventional procedure in case of complications |
| Educational purpose | Training in TUS and pleural procedures | Surgeons and respiratory physicians’ reluctance at referral | Discuss patients with MDT |
| Research purpose | Protocol development, technology improvement | Indwelled catheter management, medications | Improve home care service |

PS: pleural service. TUS: thoracic ultrasound. MDT: multidisciplinary team.

**Supplementary Figure 1:** **Number of visits performed by the ambulatory pleural service between 2018 and 2023**. 273 is the estimated number of visits in 2023 based on the visits performed in the first 3 months of the year.

**Supplementary Figure 2:** US image of a multi-loculated pleural effusion prior to thoracoscopy in non-small cell lung cancer

**Supplementary Figure 3**: subcostal chest US showing an IPC in a pleural and abdominal effusion due to ovarian cancer
